# Reply: pineal parenchymal tumors of intermediate differentiation: in need of a stringent definition to avoid confusion

**DOI:** 10.1007/s00401-024-02685-2

**Published:** 2024-02-10

**Authors:** Ramin Rahmanzade, Felix Sahm

**Affiliations:** Department of Neuropathology, German Cancer Research Center (DKFZ), University Hospital Heidelberg, and CCU Neuropathology, German Consortium for Translational Cancer Research (DKTK), Im Neuenheimer Feld 224, 69120 Heidelberg, Germany

In the comment to our recent study on Pineal Parenchymal Tumors of Intermediate Differentiation (PPTID) [3], the author raised important issues regarding the classification of pineal tumors. Indeed, the typing of tumors arising in this region needs to be revisited in a larger effort. In our cohort, we specifically focused on tumors that were, based on the current WHO classification CNS5, compatible with PPTID and had been diagnosed as such before [2]. A clinically annotated, similarly sized cohort of pineocytoma was not available, hence comparison towards this pole of the classification spectrum is limited.

However, our findings may still provide similarly strong arguments against a designation as pineocytoma as the comment argues in favour of it:

The current WHO classification includes “low proliferative/mitotic activity” as an essential criterion of pineocytoma. Including the proposed PPTID-C group, which according to our data has at least moderate proliferative activity, into pineocytoma would thus necessitate abandoning this so far decisive criterion. In fact, CNS5 has just emphasized the role of proliferative/mitotic activity over other morphological criteria compared to the revised CNS4 (summarized in [1]). Indeed, this is further supported by the wide spectrum and overlap in morphology within the cases of our study from pineocytoma to PPTID-A, B through the proposed PPTID-C.

Despite the diverging interpretation of the data available so far, we strongly agree that the classification of pineal tumors needs more insight into the biology of these tumors, since, exactly as the comment notes, the currently established and proposed types and subtypes are still enriched for specific markers and morphological features, but not defined by these alterations.
